# Probiotics May Have Beneficial Effects in Parkinson's Disease: *In vitro* Evidence

**DOI:** 10.3389/fimmu.2019.00969

**Published:** 2019-05-07

**Authors:** Luca Magistrelli, Angela Amoruso, Luca Mogna, Teresa Graziano, Roberto Cantello, Marco Pane, Cristoforo Comi

**Affiliations:** ^1^PhD Program in Clinical and Experimental Medicine and Medical Humanities, University of Insubria, Varese, Italy; ^2^Neurology Unit, Department of Translational Medicine, Interdisciplinary Research Centre of Autoimmune Diseases, Movement Disorders Centre, University of Piemonte Orientale, Novara, Italy; ^3^Biolab Research Srl, Research and Development, Novara, Italy

**Keywords:** probiotic, neuroinflammation, Parkinson's disease (PD), oxidative stress, cytokines

## Abstract

**Background:** Parkinson's disease (PD) is characterized by loss of dopaminergic neurons and intraneuronal accumulation of alpha-synuclein, both in the basal ganglia and in peripheral sites, such as the gut. Peripheral immune activation and reactive oxygen species (ROS) production are important pathogenetic features of PD. In this context, the present study focused on the assessment of *in vitro* effects of probiotic bacterial strains in PBMCs isolated from PD patients vs. healthy controls.

**Methods:** 40 PD patients and 40 matched controls have been enrolled. Peripheral blood mononuclear cells (PBMCs) were isolated and co-cultured with a selection of probiotics microorganisms belonging to the *lactobacillus* and *bifidobacterium* genus. *In vitro* release of the major pro- (Tumor Necrosis Factor-alpha and Interleukin-17A and 6) and anti-inflammatory (Interleukin 4 and 10) cytokines by PBMCs, as well as the production of ROS was investigated. Furthermore, we assessed the ability of probiotics to influence membrane integrity, antagonize the growth of potential pathogen bacteria, such as *Escherichia coli* and *Klebsiella pneumoniae* and encode tyrosine decarboxylase genes (*tdc*).

**Results:** All probiotic strains were able to inhibit inflammatory cytokines and ROS production in both patients and controls. The most striking results were obtained in PD subjects with *L. salivarius* LS01 and *L. acidophilus* which significantly reduced pro-inflammatory and increased the anti-inflammatory cytokines (*p* < 0.05). Furthermore, most strains determined restoration of membrane integrity and inhibition of *E. coli* and *K. pneumoniae*. Finally, we also showed that all the strains do not carry *tdc* gene, which is known to decrease levodopa bioavailability in PD patients under treatment.

**Conclusions:** Probiotics exert promising *in vitro* results in decreasing pro-inflammatory cytokines, oxidative stress and potentially pathogenic bacterial overgrowth. *In vivo* longitudinal data are mandatory to support the use of bacteriotherapy in PD.

## Introduction

Parkinson's disease (PD) is a common neurodegenerative disease, characterized by loss of dopaminergic neurons and intracellular accumulation of alpha-synuclein (α-syn) in the surviving neurons (1). Involvement of inflammatory mechanisms, with an imbalance between detrimental and protective immune functions (2), as well as neurotoxicity of reactive oxygen species (ROS) have been documented by several studies (3, 4). Both neuroinflammation and ROS may favor α-syn aggregation which may in turn increase pro-inflammatory cytokines and oxidative stress, thus triggering a vicious circle (5). Clinical presentation of PD is classically defined by the presence of motor symptoms such as bradykinesia, rest tremor, and rigidity. On the other hand, patients often complain of non-motor symptoms like hyposmia, constipation, pain and psychiatric conditions (e.g., anxiety, depression) that in many cases may precede the onset of clinically established disease (6, 7). Seminal work by Braak et al. hypothesized an initial aggregation of α-syn in the gut with subsequent propagation along the vagus nerve to the brain to reach the substantia nigra in the mesencephalon (8). Moreover, constipation represents a relevant symptom of PD, affecting about 70–80% of patients (9) and may precede motor symptoms by 20 years (10). The loss of enteric dopaminergic neurons determines an impairment of gastric mobility with an increased dopaminergic content and overexpression of dopaminergic receptors in the stomach (11). Furthermore, PD patients present an increased intestinal permeability and higher expression of colonic pro-inflammatory cytokines (12). Accordingly, in an α-syn overexpressing murine model of PD, gut microbiota is necessary for both microglia activation and motor impairment (13). In addition, a direct correlation between gut bacterial count and disease progression was found in PD patients (14). PD patients have a different composition of gut microbiota compared to healthy subjects (15) with reduced levels of *Prevotellaceae* and abundance of *Enterobacteriaceae* (16). *Prevotella* are in fact involved in the production of thiamine and folate, both of which are important for proper intestinal homeostasis (17). On the contrary, increased levels of *Enterobacteriaceae* have been associated to a severe PD phenotype with postural instability and gait difficulty. (16).

Of note, there is evidence that probiotics may modulate not only inflammation through cytokines production (18, 19), but also oxidative damage through a down-modulation of ROS (20). Another relevant aspect of the host-microbial interaction is the established role of infections in accelerating clinical decline in PD patients (21). In a recent investigation, focused on the clinical features and therapeutic outcomes of infected patients with or without PD, the incidence of respiratory tract and urinary tract infections was higher in PD than in age and sex-matched non-PD patients. Additionally, a longer mean hospitalization time was observed in the PD group (22). In this regard, specific probiotic strains may potentially counteract the growth of common pathogens, such as *Escherichia coli* (*E. coli*) and *Klebsiella pneumoniae (K. pneumoniae)* (23, 24). Furthermore, Van Kessel et al. recently reported that some probiotic strains produce tyrosine decarboxylase (TDC) (25). This bacterial enzyme efficiently converts levodopa to dopamine in the gut, even in the presence of human decarboxylase inhibitors or tyrosine, a competitive substrate. Accordingly, *in situ* levels of levodopa in PD patients are decreased by significant abundance of gut bacterial TDC (25). TDC genes (*tdc*) have been detected in particular in the genome of numerous bacterial species within the genera *Lactobacillus* and *Enterococcus* (26, 27). Abundance of bacterial *tdc* in stool specimens of PD patients was indeed correlated with increased daily dosage requirement of levodopa (25).

On this background, the aim of our study was to investigate the *in vitro* effects of probiotics on samples from a group of PD patients compared to healthy subjects. To do that, we assessed cytokine and reactive oxygen species (ROS) release by peripheral blood mononuclear cells (PBMCs), and restoration of artificial membrane permeability. In addition, we investigated the ability of the selected probiotics to directly inhibit *E. coli* and *K. pneumoniae*. Finally, we verified the absence of *tdc* within the genome of the selected probiotic strains.

## Patients and Methods

### Patients

We enrolled 40 patients with PD (15 women and 25 men, mean age 70 ± 8 years) and 40 age-matched healthy donors (HD, 18 women and 22 men, mean age 68 ± 7 years). PD diagnosis was performed according to the Movement Disorders Society (MDS) diagnostic criteria, e.g., when: (a) subjects presented with a parkinsonism, defined as bradykinesia, associated to rest tremor or rigidity without signs of atypical parkinsonism; (b) exclusion criteria, red flags and supportive criteria were assessed (28).

Patients were regularly followed-up at the Movement Disorder Center of Maggiore Hospital in Novara (Italy). For each patient the following parameters were considered: gender, age at onset, disease duration, Hoehn & Yahr stage (29), UPDRS III score (30), and PD therapy calculating the levodopa equivalent doses (LED) of each drug according to Tomlinson et al. (31).

Subjects with past or concomitant autoimmune disease and with a previous or ongoing immune-modulating or suppressive therapy were excluded. All subjects underwent a complete blood cell (CBC) analysis including C-Reactive Protein (CRP) and erythrocyte sedimentation rate in order to exclude both defects or activation of the immune system. All subjects were of Italian origin.

This study was approved by the local Ethics Committee (CE 65/16). Patients were included in the study after having read and signed an informed consent form for research purpose.

### Cell Cultures

Twenty milliliters of blood were drawn by venipuncture in vacuum tubes containing heparin on the same day of the clinical assessment. In order to rule out any confounding factors caused by circadian rhythm, all samples were collected at the same time of the day. Human PBMCs were isolated from heparinized blood by Healthy Donors (HD-PBMCs) and PD patients (PD-PBMCs). For cell isolation, standard techniques of dextran sedimentation and Histopaque (density = 1.077 g/cm^3^) gradient centrifugation (400 × g, 30 min, room temperature) were used. Cells were then recovered by thin suction at the interface. Isolated cells were then re-suspended in RPMI 1640 medium supplemented with 5% fetal bovine serum (FBS) and 2 mM glutamine. Cell viability (trypan blue dye exclusion) was usually >98%.

### Bacteria and Growth Conditions

Six probiotic strains (*Lactobacillus salivarius* LS01 DSM 22775, *Lactobacillus plantarum* LP01 LMG P-21021, *Lactobacillus acidophilus* LA02 DSM 21717, *Lactobacillus rhamnosus* LR06 DSM 21981, *Bifidobacterium animalis* subsp. *lactis* BS01 LMG P-21384, *Bifidobacterium breve* BR03 DSM 16604), from the Probiotical SpA collection, have been used in the present study: probiotic strains were stored in 20% glycerol at −80°C. More than 90% of the cells were alive upon thawing. Before use, microorganisms were grown in anaerobic conditions with CO_2_-generating kits (Anaerocult A; Merck, Darmstadt, Germany) overnight at 37°C in de Man-Rogosa-Sharpe (MRS) broth containing 0.05% cysteine hydrochloride, and then sub-cultured until the mid-log phase. For the enumeration of live bacteria, the BD Cell Viability Kit (BD Biosciences, Milan, Italy) were used as instructed by the manufacturer. For stimulation experiments, bacteria were suspended in RPMI-1640 medium [Invitrogen, Italy] and added to PBMCs cultures.

### Cytokine Release

Cytokine release by PBMCs was measured with an enzyme-linked immunoassay kit according to the manufacturer's instructions (ELISA Ready-SET-Go! Affymetrix eBioscience, USA). Interleukin 17A (IL-17A), tumor necrosis factor α (TNF-α), and IL-10 were assessed in both healthy controls and PD patients, whereas IL-6 and IL-4 were assessed only in PD-PBMCs (before and after probiotic stimulation). The levels of each cytokine were calculated in ρg/ml, in accordance with the manufacturer's instructions. For these experiments, HD and PD PBMCs (1 × 10^6^ cells/plate) were pre-treated for 24 h with the indicated probiotic strains in 1:1 ratio. Only HD PBMCs were treated previously with purified lipopolysaccharide (LPS) from *E. coli* 055:B5 (Sigma Chemicals, Milan, Italy) at a concentration of 10 g/mL.

### Superoxide Anion (${\text{O}}_{2}^{-}$) Production

HD- and PD-PBMCs (1 × 10^6^ cells/plate) were treated for 24 h with probiotic strains in 1:1 ratio. ${\text{O}}_{2}^{-}$ production was evaluated by the superoxide dismutase-sensitive cytochrome C reduction assay and calculated as nmol reduced cytochrome C/10^6^ cells/30 min, using an extinction coefficient of 21.1 mM. To avoid interference with spectrophotometrical recordings, cells were incubated with RPMI 1,640 without phenol red and FBS. Basal values (${\text{O}}_{2}^{-}$ production from unstimulated PBMCs) in HD were 2.2 ± 0.4 nmol reduced cytochrome C/10^6^ cells/30', and in PD patient-PBMCs were 140 ± 12 nmol reduced cytochrome C/10^6^ cells/30'. PMA is a stimulus known to induce a strong and significant respiratory burst. In line with this, PMA 10^−7^ M exposure determined a significant increase of cytochrome C levels in HD-PBMCs: 52 ± 4.5 nmol reduced cytochrome C/10^6^ cells/30′.

### Measurements of TransEpithelial Electrical Resistance (TEER)

Caco-2 cells are human colonic adenocarcinoma cells that form confluent, polarized epithelial monolayers with well-differentiated intercellular tight junctions structures. The integrity of the barrier function can be measured with TransEpithelial Electrical Resistance (TEER). TEER is an *in vitro* measurement of the movement of ions across the paracellular pathway.

A reduction in TEER may represent an early expression of cell damage and indicates that the barrier function of the intestine is decreased. Caco-2 cell lines have been extensively used over the last 20 years as a model of the intestinal barrier. The experiment was performed with an inflammatory stressor (a combination of TNF-α and IL1-ß), which is known to reduce the relative TEER of Caco-2 cells. The monolayer of Caco-2 cells was first exposed to the probiotic bacteria for 1 h, followed by exposure to the inflammatory stressor in the presence of the same probiotic bacteria, also for 1 h. After a recovery time of 24 h the TEER of the monolayer was measured. The results were compared to the TEER of a monolayer that was exposed to the stressor alone and to an unexposed sample. Cells were grown in Dulbecco's Modified Eagle Medium (DMEM) with 10% fetal bovine serum. Caco-2 (1 × 10^6^ cells/well) differentiated at 20 days were provided by the Anemocyte s.r.l. (Gerenzano, Varese, Italy). TEER was measured in each monolayer before adding 10^6^ AFU [Active Fluorescent Units were evaluated with cytofluorimetric analytical method ISO 19344:2015 (E)-IDF 232:2015 (E)] of probiotic strains onto the apical surface for 24 h prior to treatment of the basolateral medium with TNF-α and IL1-ß (10 ng/ml; Thermo Scientific, USA). It was determined that 10^6^ AFU of probiotic strains did not cause deleterious effects on epithelial cells over the time-course of the experiments and that the medium used in this experiment did not cause bacterial overgrowth.

### Spot-on Lawn Antimicrobial Assay/Agar Spot Antimicrobial Assay

The antimicrobial activity against *E. coli* and *K. pneumoniae* was assessed according to the protocol described by Santini C. (32). Briefly, 5 μl of probiotic overnight fresh cultures with an optical density (OD) at 600 nm close to 1 were spotted on the surface of MRS agar plates and incubated anaerobically for 5 h at 37°C to allow strain development (spot). The *E. coli* ATCC 8739 strain or *K. pneumoniae* ATCC 13883 strain was inoculated in Brain Heart Infusion (BHI) soft agar and dispensed onto spot plates. When the top agar was solid, the plates were inverted and incubated in conditions of anaerobiosis at 37°C for 48 h. At the end of incubation, plates were examined for the appearance of clear zones showing the antagonistic activity. The plate inhibition technique experiments were carried out on triplicates, and the mean values of growth inhibition zones around the disks were measured using a ruler (mm) and recorded.

### Search for Tyrosine Decarboxylase (TDC) Genes

To identify whether the genomes of the probiotic strains employed encoded *tdc*, the TDC protein sequence (EOT87933) from *Enterococcus faecalis* v583 was used as a query along with *E. faecalis* v583 as a positive control using the BLAST program of PATRIC suite (www.patricbrc.org). *E. faecalis* V583 TDC protein sequence (NCBI accession: EOT87933) was downloaded in FASTA format. PATRIC suite was used for the Annotation of the six bacterial strains using the RAST tool kit encoded within the software. Annotated genomes were grouped together and BLASTed against the TDC protein sequences.

### Statistical Analysis

Results are expressed as the means ± SEM of duplicate measures determined in three independent experiments. Differences between unstimulated and stimulated samples were tested using the *t* test with GraphPad Prism 6.0 software. Correlations between probiotic strain and clinical-demographic variables were calculated using Spearman test. Values of *p* < 0.05 were considered significant.

## Results

### Cytokine Release

**Figure 1:**
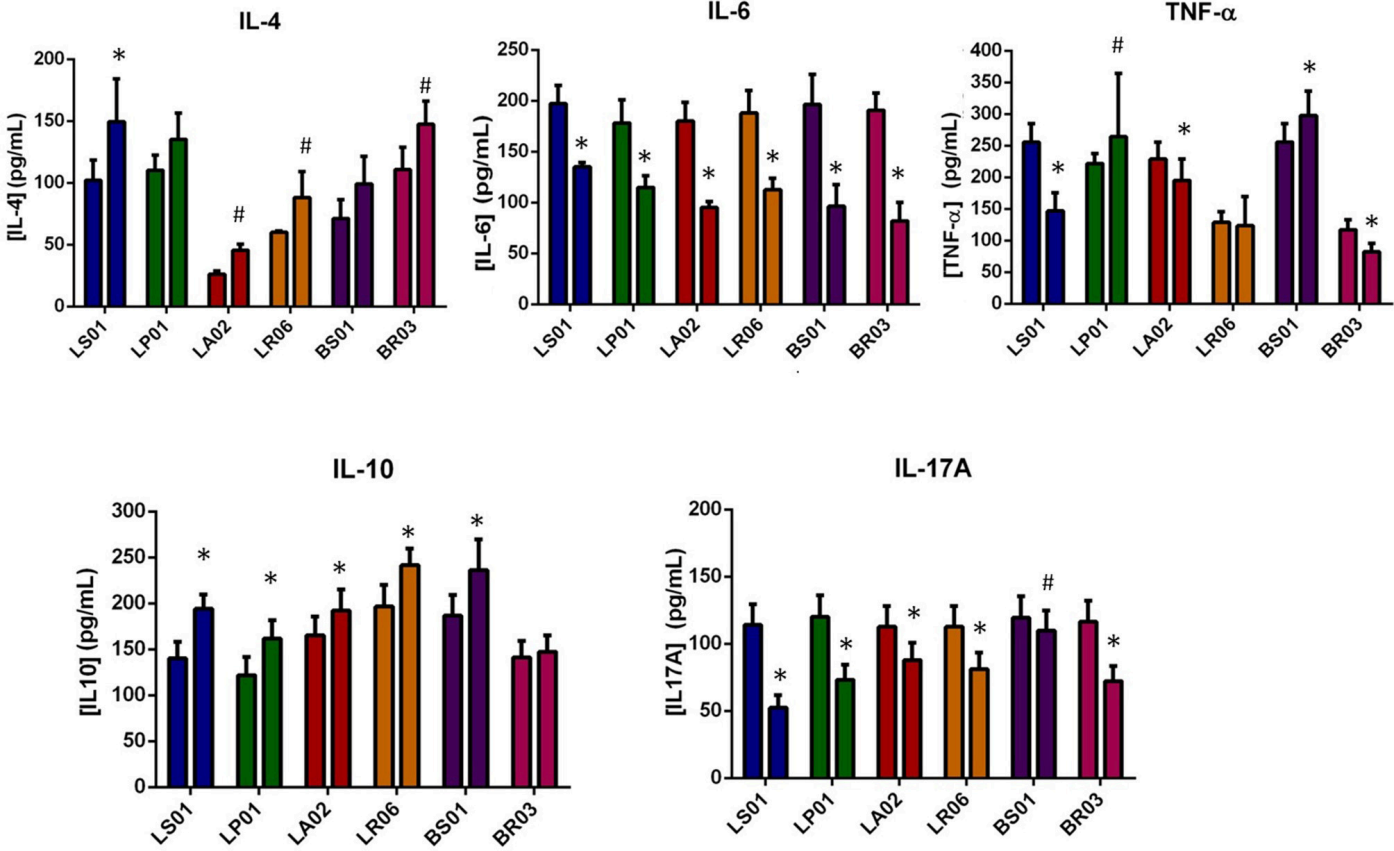
Modulation of cytokine production by probiotic strains. For each probiotic strain the first column indicates the baseline values, while the second after probiotic administration. *p < 0.001; #*p* < 0.05 post vs. pre-exposure from PD-PBMCs.

IL-10 and IL-4 are important cytokines in the regulation of immune responses, counterbalancing the pro-inflammatory effects of TNF-α, IL-6 and IL-17A. Cytokines modulation by different probiotic strains was measured in PBMCs from both PD patients and healthy donors (Table 1 and Figure 1; Table S1). In PD-PBMCs, most probiotic strains determined a statistically significant reduction of pro-inflammatory cytokines production (TNF-α, IL-6, and IL-17A) and an increase of the anti-inflammatory IL-4 and IL-10. The most striking results were obtained with LS01 (TNF-α: baseline 255.52 ± 29.55 pg/ml, after stimulus 146.69 ± 28.67 pg/ml, *p* < 0.001; IL-6 baseline 197.2 ± 18.2 pg/ml, after stimulus 132.5 ± 4.2 pg/ml, *p* < 0.001; IL17-A baseline 114.08 ± 15.41 pg/ml, after stimulus 52.48 ± 9.25 pg/ml, *p* < 0.001; IL-4 baseline 102.00 ± 16.4 pg/ml, after stimulus 149.6 ± 34.7 pg/ml, *p* < 0.001; IL-10: baseline 140.12 ± 18.01 pg/ml, after stimulus 194.24 ± 15.42 pg/ml, *p* < 0.001) and LA02 (TNF-α: baseline 228.90 ± 26.89 pg/ml, after stimulus 195.15 ± 33.52 pg/ml, *p* < 0.001; IL-6 baseline 180.2 ± 92.5 pg/ml, after stimulus 92.5 ± 5.9 pg/ml, *p* < 0.001; IL17-A baseline 112.81 ± 15.28 pg/ml, after stimulus 87.99 ± 12.80 pg/ml, *p* < 0.001; IL-4 baseline 26.3 ± 2.5 pg/ml, after stimulus 45.6 ± 4.9 pg/ml, *p* < 0.05; IL-10: baseline 165.33 ± 20.53 pg/ml, after stimulus 192.18 ± 23.22 pg/ml, *p* < 0.001). The remaining data, including all *p* values, are shown in Table 1.

**Table 1:** Modulation of cytokines production by the probiotic strains in PD patients.

**Probiotic strain**	**IL10**		**TNF-α**		**IL17-A**		**IL4**		**IL6**	
	**Mean**	**SEM**	**Mean**	**SEM**	**Mean**	**SEM**	**Mean**	**SEM**	**Mean**	**SEM**
LS01	Baseline	140.12	18.01	255.52	29.55	114.08	15.41	102.00	16.4	197.2	18.2
	After stimulus	194.24	15.42	146.69	28.67	52.48	9.25	149.6	34.7	135.2	4.2
	*P*	**<0.001**		**<0.001**		**<0.001**		**<0.01**		**<0.001**	
LP01	Baseline	121.81	20.17	221.80	16.18	120.04	16.00	110.2	12.3	178.2	22.8
	After stimulus	161.70	20.17	264.10	100.41	73.22	11.32	135.2	21.3	114.9	11.4
	*P*	**<0.001**		**0.01**		**<0.001**		n.s.		**0.01**	
LA02	Baseline	165.33	20.53	228.90	26.89	112.81	15.28	26.3	2.5	180.2	18.2
	After stimulus	192.18	23.22	195.15	33.52	87.99	12.80	45.6	4.9	92.5	5.9
	*P*	**<0.001**		**<0.001**		**<0.001**		**<0.05**		**<0.001**	
LR06	Baseline	196.71	23.67	128.90	16.89	112.81	15.28	60.00	1.15	188.0	22.30
	After stimulus	241.63	18.16	123.54	46.35	81.22	12.12	88.2	21.1	112.5	11.4
	*P*	**<0.001**		n.s		**<0.001**		**<0.05**		**<0.001**	
BS01	Baseline	186.71	22.67	255.74	29.57	119.44	15.94	71.2	15.3	196.4	29.6
	After stimulus	236.34	33.63	297.82	38.78	109.88	14.99	99.2	22.4	96.3	21.4
	*P*	**<0.001**		**<0.001**		**0.007**		n.s		**<0.001**	
BR03	Baseline	141.12	18.11	117.19	15.72	116.43	15.64	110.7	18.11	190.8	16.7
	After stimulus	147.38	17.74	82.30	13.14	72.19	11.22	147.38	18.7	81.9	18.1
	*P*	n.s.		**<0.001**		**<0.001**		**<0.05**		**<0.001**	

*All data are expressed in pg/ml; n.s., not statistically significant*.

### Superoxide Anion (${\text{O}}_{2}^{-}$) Production

**Figure 2:**
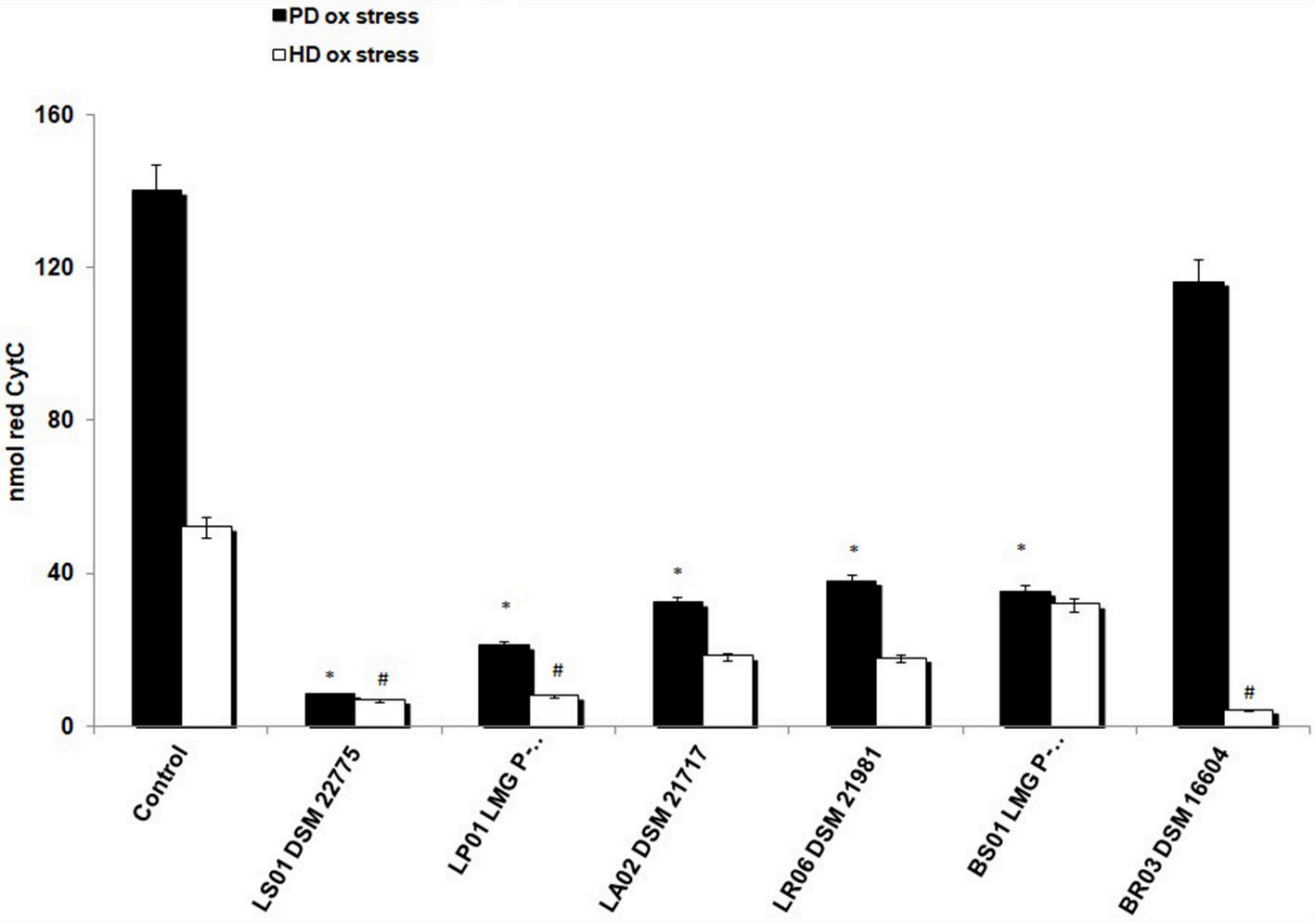
Reduction of superoxide anion (${\text{O}}_{2}^{-}$) production. *p < 0.01 post vs. pre-exposure from PD-PBMCs. #*p* < 0.05 post vs. pre-exposure from PMA-exposed HD-PBMCs.

First, we found a statistically significant difference in baseline O^2−^ production from unstimulated PBMCs between patients and controls. In fact, reduced cytochrome C in HD was 2.2 ± 0.4 nmol vs. 140 ± 12 nmol/10^6^ cells/30' in PD-PBMCs (*p* < 0.001). To test the antioxidant effects of probiotics on HD-PBMCs we induced a toxic condition using PMA, a stimulus known to determine a strong and significant respiratory burst. Consistently, PMA exposure in HD-PBMCs increased levels of reduced cytochrome c (52 ± 4.5 nmol). As depicted in Figure 2, after exposure to probiotic strains, we found an overall decrease of ${\text{O}}_{2}^{-}$ production in unstimulated PBMCs from PD patients and in PMA-stimulated PBMCs from HD. In detail, LS01, LP01, LA02, LR06, BS01 caused a robust decrease of ${\text{O}}_{2}^{-}$ from PD-PBMCs (*p* < 0.01 post vs. pre-exposure). A weaker, though statistically significant, effect was obtained from PMA-stimulated HD-PBMCs after exposure to LS01, LP01, BR03 (*p* < 0.05 post vs. pre-exposure).

### TEER Evaluation

Three strains (LP01, LR06, and BR03) provided higher protection of epithelial cells against the cytokine-induced barrier dysfunction (*p* < 0.001), whereas three others (LS01, LA02, and BS01) had a lower, though still significant, effect (*p* < 0.05). The TEER ratio was measured before adding the bacterial inoculum (CTR) and in a damage-tissue model and after addition and incubation of the probiotic strains on Caco-2 monolayer. The results are shown in Table 2 and Figure S1.

**Table 2:** TERR evaluation using different probiotic strains.

	**LA02**	**LP01**	**LR06**	**LS01**	**BR03**	**BS01**
Baseline integrity	100%	100%	100%	100%	100%	100%
Integrity after damaging stimulus	40%	40%	40%	40%	40%	40%
Integrity after probiotic strain	91%	100%	100%	81%	94%	84%
*P* value	<0.05	<0.001	<0.001	<0.05	<0.05	<0.001

### Inhibition of *E. coli* and *K. pneumoniae*

As shown in Table 3, most probiotic strains showed a robust inhibitory capacity against the target pathogen strains *E. coli* and *K. pneumoniae* involved in the comorbidities of PD. Particularly, LP01 and LR06 exerted the highest inhibition. Moreover, such inhibition was not observed when a negative control (MRS acidified at pH 4.3 but without any probiotic strains; data not shown) was tested, thus confirming the specificity of the detected antagonistic activity.

**Table 3:** Inhibition of bacterial overgrowth exerted by probiotic strains.

**Probiotic strain [inhibition (mm)]**	***E. coli***		***K. pneumoniae***	
	**Mean**	**SEM**	**Mean**	**SEM**
LS01	0.43	0.15	0.58	0.17
LP01	0.57	0.14	1.42	0.56
LA02	0.30	0.11	0.23	0.10
LR06	0.48	0.10	0.63	0.37
BS01	0.01	0.004	0.11	0.05
BR03	0.01	0.004	0.15	0.01

*Results are expressed in mm*.

### Tyrosine Decarboxylase (TDC) Genes

Only *E. faecalis* V583 gave an Identity value equal to 100% with a Query Cover of 100%, whilst the other probiotic strains scored below 35% of Identity, thus excluding the expression of a tyrosine decarboxylase activity within the six tested probiotic strains (data not shown).

### Clinical Analysis

#### Demographic Results

Mean age at PD onset was 65 ± 8 years. In detail, at study enrollment, 4 patients were drug naïve, 26 were taking levodopa (one of them was on Duodopa) and 10 were taking dopaminergic treatment other than levodopa (dopamine agonists and MAO inhibitors). Mean LED was 469.9 mg/day ± 354. Mean UPDRS III was 12.45 ± 6.9 points.

#### Gender

Comparing responses to probiotics in relation to gender, we found a statistically significant difference between male and female PD patients in ROS production from PBMCs. The effect of LR06 was more pronounced in samples from male vs. female donors (81 and 60% reduction compared to baseline levels, respectively; *p* < 0.05; Table 4). On the contrary, BS01 was more effective in samples from female than male patients (84 and 69% reduction compared to baseline levels, respectively; *p* < 0.05; Table 4).

**Table 4:** Comparison of ROS production after each probiotic stimulation between female and male patients.

	**LA02**	**LP01**	**LR06**	**LS01**	**BR03**	**BS01**
Female	0.29	0.16	0.40	0.06	0.82	0.16
Male	0.20	0.14	0.19	0.06	0.83	0.31
*p* value	0.17	0.67	**0.005**	0.97	0.97	**0.03**

*Data are expressed as the value after probiotic strain exposure from a baseline conventionally established as 1. The statistically significant differences are shown in bold*.

#### Disease Duration

**Figure 3:**
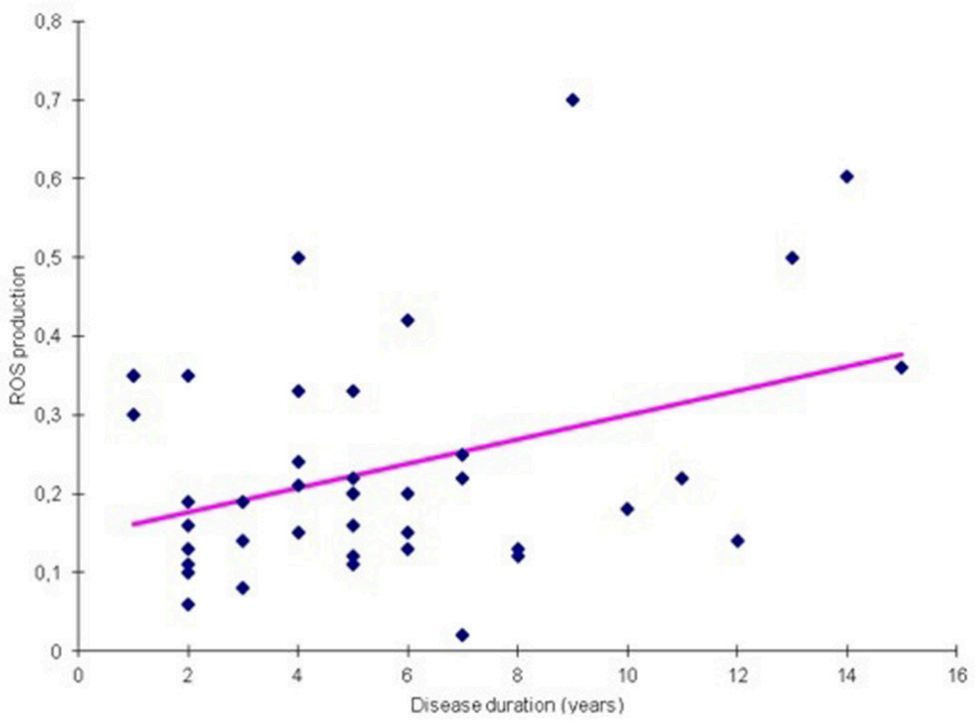
Correlation between ROS production and disease duration for probiotic LA02 (*p* = 0.002).

We analyzed ROS and cytokine levels from PD-PBMCs exposed to different probiotic strains in relation to disease duration. We found that LA02 provided a robust anti-oxidant effect, which decreased significantly in samples of patients with longer disease duration (rho = 0.22, *p* < 0.05 Table 5, Figure 3). Such correlation was not detected with other strains. Furthermore, we found no other statistically significant correlation between the effect of the different strains and the remaining clinical variables (H&Y stage, UPDRS score, LED, data not shown).

**Table 5:** Correlations between probiotic strains modulation of ROS production and clinical characteristics of PD patients.

**Probiotic strain**	**Disease duration**	**H&Y stage**	**UPDRS III score**	**LED**
LA02 (rho; p)	**0.22; 0.002**	0.18; 0.94	0.04; 0.55	0.03; 0.22
LP01	0.05; 0.19	−0.03; 0.53	0.07; 0.53	−0.01; 0.60
LR06	0.08; 0.88	−0.08; 0.83	−0.16; 0.40	0.06; 0.80
LS01	0.17; 0.61	0.25; 0.55	0.28; 0.63	0.05; 0.12
BR03	−0.01; 0.83	0.08; 0.84	−0.10; 0.88	0.01; 0.73
BS01	−0.05; 0.45	0.32; 0.21	0.06; 0.33	0.14; 0.48

*Data are expressed as the value after probiotic strain exposure from a baseline conventionally established as 1. The statistically significant differences are shown in bold*.

## Discussion

In this study we showed that probiotic strains modulate the release of cytokines and ROS by PBMCs of PD patients and healthy controls. Particularly, *L. salivarius* (LS01) and *L. acidophilus* (LA02) showed the best profiles in PD-PBMCs, being able to significantly decrease all the pro-inflammatory cytokines and increase the anti-inflammatory ones. The same strains were also able to significantly reduce ROS production in both PD and HD-PBMCs. In addition, all the tested probiotic strains restored epithelial damage in Caco-2 cells. Finally, the tested probiotic strains exerted a robust capacity of inhibiting *E. coli* and *K. pneumoniae*. These Gram-negative bacteria are frequently detected in blood cultures of septic PD patients (22). Such antagonistic activity might be ascribed to the active bacteriocins secreted by probiotic strains. Probiotics have been studied in PD for their potential symptomatic effect on constipation (33) and probiotic strains BS01, LP01, and BR03 have shown improvement in constipation and associated symptoms in healthy adults (34) and more interestingly in chronically constipated elderly (35). However, despite the great interest that recently arose around the gut-brain axis in health and disease, our study is the first to specifically address the effect of probiotics on mediators of inflammation and oxidative damage in PBMCs of PD patients. On the other hand, experimental evidence on the anti-inflammatory and anti-oxidative effects of probiotics is rapidly growing. *L. plantarum* displayed the capacity of decreasing the histopathological damages in a murine model of Alzheimer's disease (AD) leading to increased production of acetylcholine with consequent clinical improvement (36). Accordingly, it was shown that probiotics administration was effective in modulating cognitive functions in a group of AD patients (37). Probiotics may also be helpful in other contexts of neuroinflammation such as post-traumatic stress disorder, amyotrophic lateral sclerosis and cognitive dysfunction after surgery (38–40). Of note, in a murine model of stress, modification of gut microbiota provided both behavioral and immunological beneficial effects (41).

Altogether, such findings suggest that probiotics may represent a promising strategy to counteract the detrimental immune activation that takes place in PD. In fact, work by independent groups is indicating that peripheral and central immune responses are strictly interconnected in PD and the study of such mechanisms may provide relevant advances in both diagnostic and therapeutic areas (42, 43). An important breakthrough in this area was the demonstration of the antigenic role of α-syn on peripheral T cells: such epitopes can in fact drive T helper and cytotoxic responses in PD patients (44). Moreover, recent work by Kustrimovic et al. showed that PD patients display a predominance of Th1 mediated responses compared to healthy controls (45). A pro-inflammatory profile, with increased production of IL-1α, IL-1β, and CXCL8 was also detected in stool samples of PD patients, further supporting the involvement of intestinal immunity in PD (46). The results of the present study support the concept of a predominantly pro-inflammatory environment in the periphery, since pro-inflammatory cytokines production was significantly increased in PD patients vs. controls. Notably, the probiotic strains tested in our *in vitro* experiments were able to counterbalance such pro-inflammatory response. Our results also suggest the involvement of IL17A in PD: patients present in fact higher levels of IL17A than healthy controls. The role of IL17 producing T helper cells (Th17) in the context of neurodegeneration has not yet been completely elucidated (47). There is evidence showing that Th17 cause cell death in a human iPSC-based model of PD and also evidence that Th17 can be induced and regulated by the intestinal microbiota (48, 49).

Our study has indeed some limitations: sample size is relatively small, and the cross-sectional design suggests caution in the interpretation of results. Furthermore, our data derive from *in vitro* experiments, which might not reflect precisely the complex pathophysiological dynamics of PD and should therefore be reproduced *in vivo*. Possible strategies may involve a study on an animal model of PD, or alternatively the direct evaluation of the clinical and biological effects of probiotics administration in PD patients. In both cases, a longitudinal study in which biomarkers and clinical findings are collected before and after probiotics administration would likely provide important responses.

Our data also suggest that the effect of probiotics might be different with respect to disease stage and gender. Accordingly, we found that LA02 provided a down-modulation of ROS that was more pronounced in the early stages of disease. These data need further confirmation since previous studies did not detect any correlations between clinical or biological variables and disease stage in PD patients treated with probiotics (46). Moreover, LR06 and BS01 displayed different anti-inflammatory and anti-oxidant activities in PBMCs from male compared to female PD patients. Of note, it was previously reported that microbiota composition may differ between male and female subjects and that this in turn may influence immune functions (50). One last open question regards the relationships between probiotics, peripheral immune function and dopaminergic therapy, especially considering that, to date, the influence of PD treatment on peripheral immunity is still controversial (51–53). Definite answers to this question, as well as to whether and how probiotic administration should be personalized, will likely come from longitudinal *in vivo* studies.

Overall, our preliminary findings suggest a potential role for probiotic strains in modulating inflammation and oxidative stress and protecting the epithelium from gut permeability. Further relevant findings include a possible inhibitory effect against *E. coli* and *K. pneumoniae*, which might be exerted without interfering with levodopa levels.

## Ethics Statement

This study was approved by the local Ethics Committee (CE 65/16). Patients were included in the study after having read and signed an informed consent form for research purpose.

## Author Contributions

All authors contributed to manuscript revision, read and approved the submitted version. CC, MP, LMo, and RC contributed to manuscript revision. CC, MP, LMa, and AA contributed to conception and design of the study. LMa and AA organized the database, performed the statistical analysis, wrote the first draft of the manuscript. MP, TG, and AA contributed acquisition, analysis or interpretation of data.

### Conflict of Interest Statement

AA, LMo, TG, and MP were employed by company Biolab Research srl, Novara, Italy. The remaining authors declare that the research was conducted in the absence of any commercial or financial relationships that could be construed as a potential conflict of interest.
