# Supplementary material for: Probiotics May Have Beneficial Effects in Parkinson's Disease: In vitro Evidence
Source: Front Immunol. 2019 May 7;10:969. doi: 10.3389/fimmu.2019.00969 (PMC6513970; doi:10.3389/fimmu.2019.00969)
Supplement: Supplementary file 1 [file Table_1.DOCX]

**Table S1**: Modulation of cytokines production by the probiotic strains in healthy controls.

| Probiotic strain | | IL-10  Mean SEM | | TNF-α  Mean SEM | | IL-17A  Mean SEM | | |
| --- | --- | --- | --- | --- | --- | --- | --- | --- |
| LS01 | Baseline | 39.12 | 5.41 | 55.52 | 6.38 | | 14.08 | 3.12 |
|  | LPS | 151.66 | 6.98 | 150.6 | 8.89 | | 95.09 | 19.61 |
|  | After stimulus | 139.09 | 6.27 | 246.69 | 45.88 | | 11.25 | 3.06 |
| LP01 | Baseline | 121.81 | 13.38 | 21.80 | 3.46 | | 20.04 | 3.08 |
|  | LPS | 356.26 | 25.63 | 479.09 | 40.85 | | 90.09 | 9.87 |
|  | After stimulus | 412.43 | 17.43 | 964.10 | 11.25 | | 7.05 | 0.71 |
| LA02 | Baseline | 95.33 | 14.89 | 28.90 | 4.71 | | 12.81 | 1.91 |
|  | LPS | 513.74 | 75.64 | 194.63 | 20.70 | | 51.75 | 2.43 |
|  | After stimulus | 631.43 | 77.74 | 295.15 | 20.59 | | 11.29 | 1.45 |
| LR06 | Baseline | 96.71 | 14.56 | 28.90 | 4.71 | | 12.81 | 1.91 |
|  | LPS | 200.77 | 27.44 | 194.63 | 20.70 | | 51.75 | 2.43 |
|  | After stimulus | 183.82 | 30.90 | 423.54 | 20.53 | | 24.21 | 3.27 |
| BS01 | Baseline | 96.71 | 14.56 | 55.74 | 8.99 | | 19.44 | 2.38 |
|  | LPS | 200.77 | 27.44 | 407.22 | 43.97 | | 82.74 | 9.35 |
|  | After stimulus | 223.16 | 24.50 | 347.82 | 33.58 | | 33.83 | 8.46 |
| BR03 | Baseline | 61.12 | 8.42 | 17.19 | 2.66 | | 16.43 | 2.76 |
|  | LPS | 213.32 | 24.92 | 133.03 | 4.91 | | 57.42 | 5.94 |
|  | After stimulus | 345.21 | 16.71 | 91.35 | 12.97 | | 10.69 | 0.44 |
