# Supplementary figures and images for: Probiotics May Have Beneficial Effects in Parkinson's Disease: In vitro Evidence
Source: Front Immunol. 2019 May 7;10:969. doi: 10.3389/fimmu.2019.00969 (PMC6513970; doi:10.3389/fimmu.2019.00969)

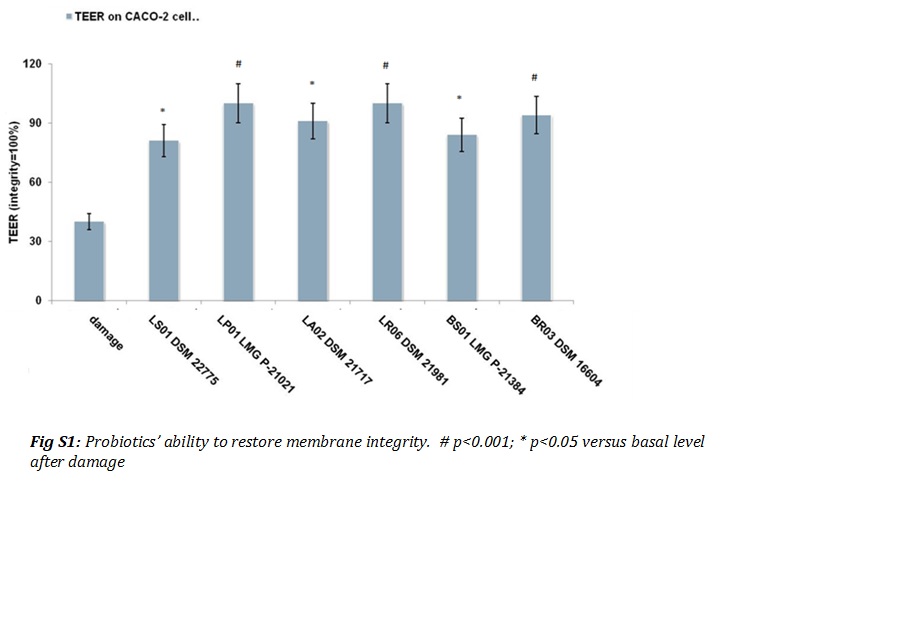

Supplement: Supplementary file 2 [file Image_1.JPEG]
